# A computational study to model the effect of electrode-to-auditory nerve fiber distance on spectral resolution in cochlear implant

**DOI:** 10.1371/journal.pone.0236784

**Published:** 2020-08-03

**Authors:** Hyejin Yang, Jong Ho Won, Inyong Choi, Jihwan Woo

**Affiliations:** 1 Department of Biomedical Engineering, School of Electrical Engineering, University of Ulsan, Ulsan, Republic of Korea; 2 Division of ENT, Sleep Disordered Breathing, Respiratory, and Anesthesia, Office of Product Evaluation and Quality, Center for Devices and Radiological Health, US Food and Drug Administration, Silver Spring, MD, United States of America; 3 Department of Communication Sciences and Disorders, University of Iowa, Iowa City, IA, United States of America; Universidad de Chile, CHILE

## Abstract

Spectral ripple discrimination (SRD) has been widely used to evaluate the spectral resolution in cochlear implant (CI) recipients based on its strong correlation with speech perception performance. However, despite its usefulness for predicting speech perception outcomes, SRD performance exhibits large across-subject variabilities even among subjects implanted with the same CIs and sound processors. The potential factors of this observation include current spread, nerve survival, and CI mapping. Previous studies have found that the spectral resolution reduces with increasing distance of the stimulation electrode from the auditory nerve fibers (ANFs), attributable to increasing current spread. However, it remains unclear whether the spread of excitation is the only cause of the observation, or whether other factors such as temporal interaction also contribute to it. In this study, we used a computational model to investigate channel interaction upon non-simultaneous stimulation with respect to the electrode–ANF distance, and evaluated the SRD performance for five electrode–ANF distances. The SRD performance was determined based on the similarity between two neurograms in response to standard and inverted stimuli and used to evaluate the spectral resolution in the computational model. The spread of excitation was observed to increase with increasing electrode–ANF distance, consistent with previous findings. Additionally, the preceding pulses delivered from neighboring channels induced a channel interaction that either inhibited or facilitated the neural responses to subsequent pulses depending on the electrode–ANF distance. The SRD performance was also found to decrease with increasing electrode–ANF distance. The findings of this study suggest that variation of the neural responses (inhibition or facilitation) with the electrode–ANF distance in CI users may cause spectral smearing, and hence poor spectral resolution. A computational model such as that used in this study is a useful tool for understanding the neural factors related to CI outcomes, such as cannot be accomplished by behavioral studies alone.

## 1. Introduction

A cochlear implant (CI) is a neuroprosthesis that partially restores functional hearing via electrical stimulation in patients with severe-to-profound sensorineural hearing loss. Because the perception of sound is based on the tonotopicity of the cochlea, a CI uses an intra-cochlear electrode array to stimulate the auditory nerve fibers (ANFs) corresponding to each characteristic frequency. The ability to resolve complex spectral patterns is therefore an important factor of speech perception in CI recipients [1, 2]. The spectral resolution of electric hearing has thus been widely evaluated by spectral ripple discrimination (SRD) tests [3]. Listeners are asked to discriminate between two rippled noise stimuli with reversed positions of their frequency peaks. Although SRD performance has been shown to significantly correlate with speech perception in CI users in several studies, the SRD performance variability among subjects is often large [4–7]. Across-subject SRD performance variability may be affected by the specific CI device (e.g., implant type [8], sound processor, or electrode design [9]), biological factors (e.g., residual hearing [10], shape of the cochlea, or electro-neural interface [11, 12]), etiology [13], or cognitive factors [14]. Controlling all these factors can be challenging in a clinical setting and using a computational model is helpful for investigating the patterns of their effects on neural excitation.

In a CI, electric stimulation is delivered through channels formed by the intra electrode array. Channel interaction and spread of excitation are thus induced, even though the CI electrodes are non-simultaneously activated, resulting in less precise neural responses with negative effects on speech perception by the subject [15, 16]. In the case of a two-pulse paradigm, channel interaction due to non-simultaneously activated electrodes tends to occur in two ways. The first involves the forward masking effect, whereby the neural response to the subsequent pulse may be depressed, possibly because of the refractoriness of the neural response [17, 18]. In the second way, the neural response to the second pulse is facilitated by the first response, and this may cause temporal integration [19, 20].

However, the manner the current delivered from the neighboring channels affects the spectral resolution in CI users remains unclear. Analyzing the effect of redundant current on the neural response and the associated spectral benefits in CI users is important. Accordingly, this study aims to investigate the effect of channel interaction on the SRD performance by observing the temporal response pattern using a computational model. The model responses to the changes in changes electrode-to-ANF distance were compared with the responses of CI users to gain a general understanding of large across-subject variabilities in SRD performance. We found that the inhibition and facilitation of the neural responses are affected by the extent of the variation in current spread depending on the electrode–ANF distance. We further found that poor SRD performance results from the facilitation of neural responses by a large electrode–ANF distance, rather than from the inhibition of neural responses by a small electrode–ANF distance.

## 2. Materials and methods

**Fig 1:**
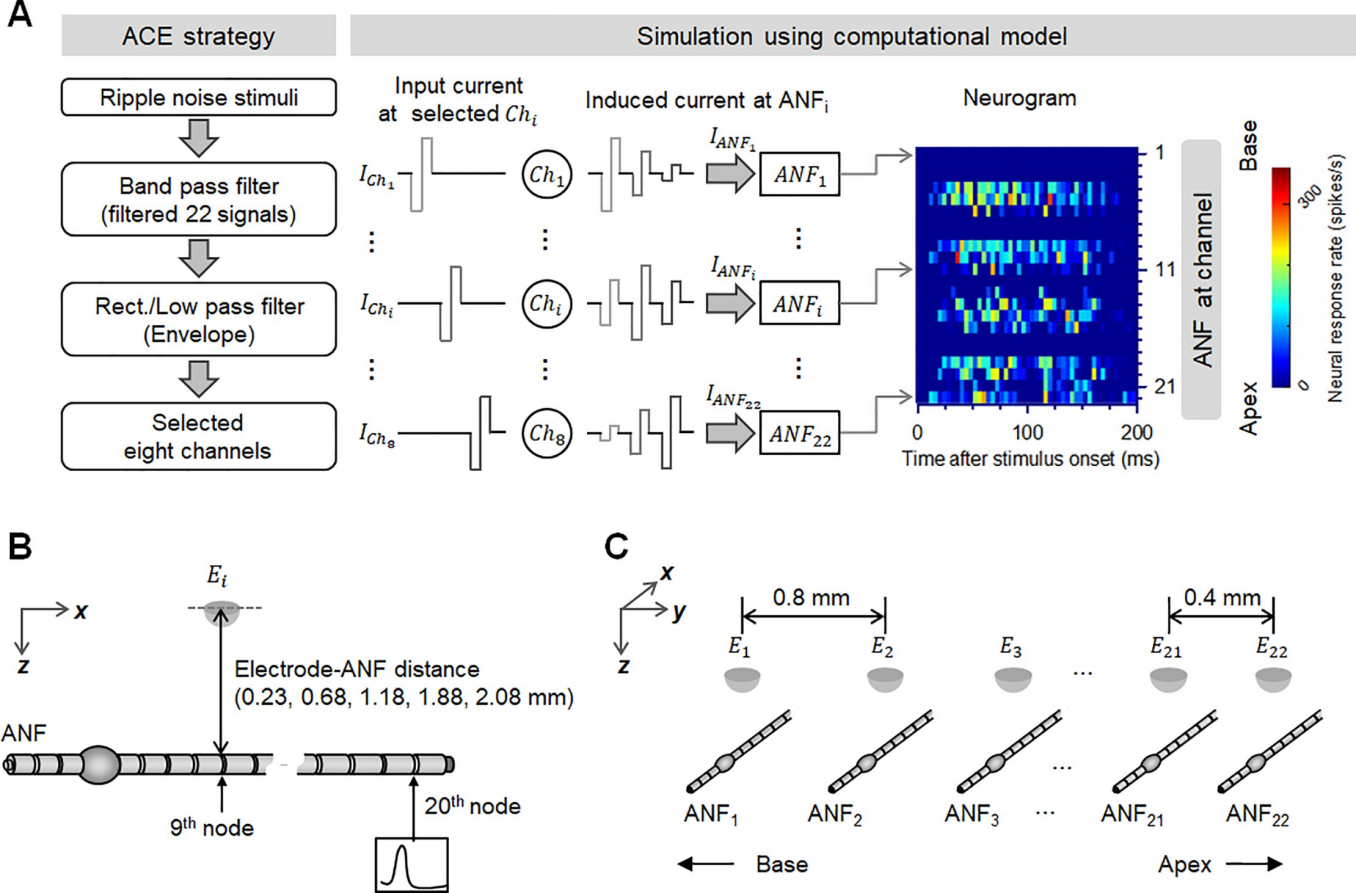
Overview of the simulation using a computational model. (A) Procedure for generating a neurogram and an example neurogram for standard spectral ripples with a ripple density of 1.0 ripples per octave (r/o). (B) Geometry of the computational model for a single electrode and ANF. (C) Electrode–neural interface with 22 electrodes and 22 ANFs. See the materials and methods section for a detailed description of each part.

Fig 1 illustrates the procedure used to simulate SRD with the aid of a computational model in this study. The spectral ripple stimuli were processed into electrical pulses using the advanced combination encoder (ACE) strategy [21], which is standard for commercial CI devices. The current level of each electric pulse at the *i*th ANF (ANF_*i*_) was determined by the distance between ANF_*i*_ and the active electrode (see Section 2.3). Finally, a neurogram representing the neural activities at each ANF_*i*_ over time was generated. The geometries of the model used in this study are shown in Fig 1B and 1C. Fig 1B shows the position of an electrode and the ANF model, as developed by Woo et al. [22], in the *x–z* plane. A hemispherical electrode of radius 0.18 mm was located on the 9th active node to stimulate an ANF, while the ANF response was recorded at the 20th active node. The electrode–neuron interface of the model is indicated in Fig 1C. The details of the neurogram generation process are presented in Sections 2.1–2.4.

### 2.1 Rippled noise stimuli

**Fig 2:**
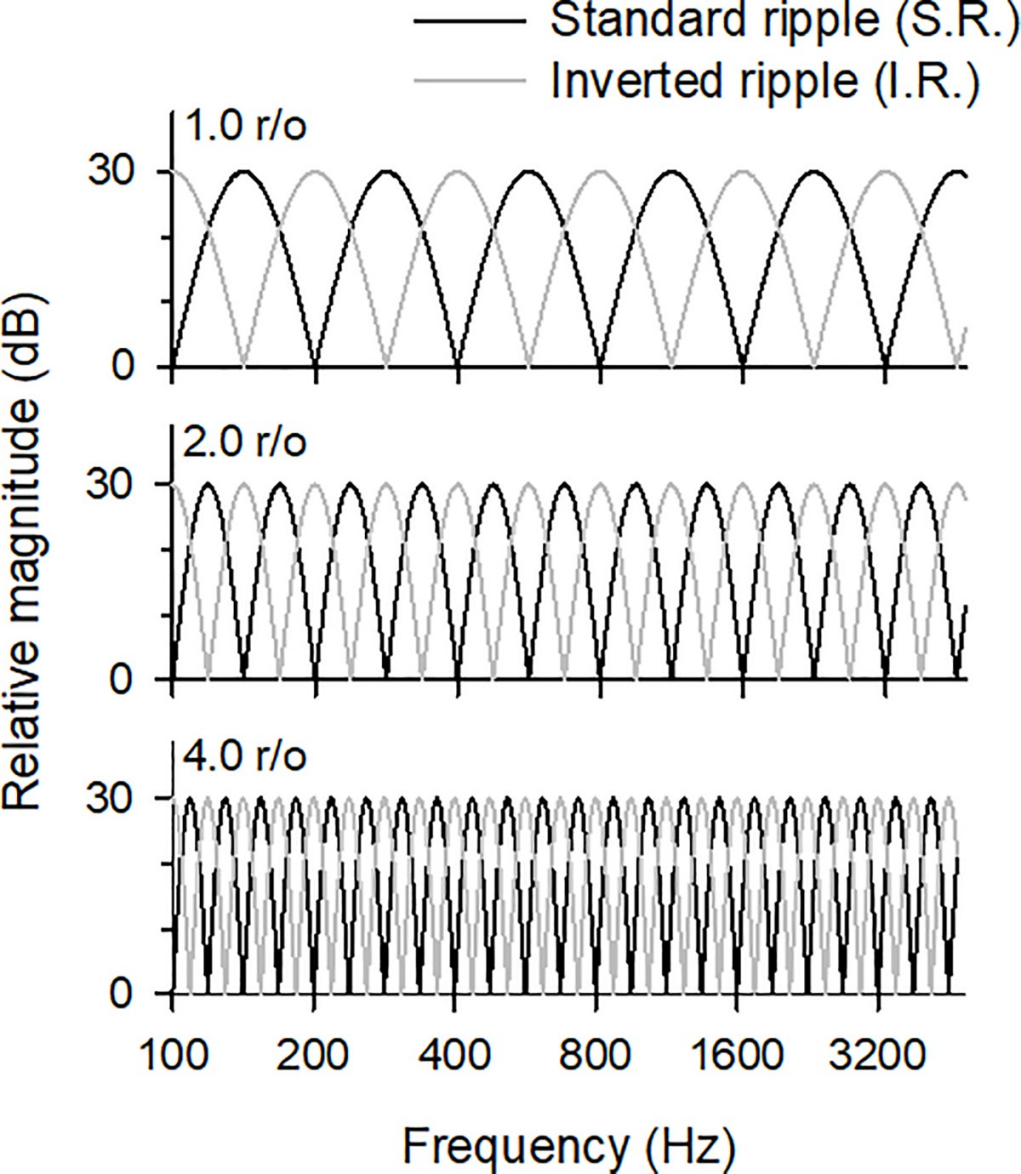
Spectra of the standard and inverted ripple noises with frequencies of 100–5000 Hz and ripple densities of 1.0, 2.0, and 4.0 r/o.

The rippled noise stimuli used to evaluate SRD performance [6] were generated from white noise produced by combining pure tones with 200 frequency components within 100–5,000 Hz using a random phase. The power spectrum was determined by a full-wave sinusoidal envelope with a peak-to-valley ratio of 30 dB. The power spectrum phase of the standard ripple noise stimuli was set to zero radians, while the inverted ripple noise stimuli had a reversed phase (*π*/2) (see Fig 2). The ripple peaks were equally spaced on a logarithmic frequency scale. The ripple densities were set to 1.0, 2.0, and 4.0 r/o, representing conditions ranging from easy to difficult as reported by CI users [6]. Fig 2 shows the spectra of the standard and inverted ripple stimuli for the three ripple densities considered in this study. The frequency positions of the peaks and valleys of the two rippled noise stimuli are interchanged, with the ripple noise stimuli of higher ripple densities consistent with the narrower ripple spacing. The stimuli were applied for a total duration of 200 ms with a ramp of 50 ms rise/fall time.

### 2.2 Cochlear implant sound processing

The acoustic rippled noise stimuli were processed by a pre-emphasis filter, band-pass filters with 22 frequency bands, and envelope detection. Eight channels with amplitudes within the 22 bands were chosen, and biphasic pulses (cathodic-first, 25 μs/phase) were modulated based on the amplitude of each of the eight selected channels using the ACE sound coding strategy. The high- and low-frequency channels were mapped to the basal and apical electrodes, respectively, which for the cochlear devices are numbered from 1 (most basal electrode) to 22 (most apical electrode). The stimulation rate was set to 900 pulses per second per channel (pps/ch) which is typically used in clinics. A self-written custom MATLAB software was used to implement all the procedures and simulate the ACE strategy. A detailed description of the stimuli generation using an ACE strategy is provided by Yang et al. [23]. The electrical dynamic range for the simulation was determined using an I/O function that described the response rate within the onset time window (0–12 ms); this covered refractory periods of 4 ms [24, 25] as a function of the 900-Hz pulse train level. The threshold (T) and comfortable (C) current levels were set to 10%–90% of the input/output (I/O) function, comparable to the psychophysical loudness scaling approach generally used in clinics to set the levels of T (level 1 out of 10) and C (level 9 out of 10) [26].

### 2.3 Modeling of auditory nerve fibers and applied electric stimulation

The CI configuration of the model was based on a Nucleus CI24 implant with an inter-electrode distance range of 0.4–0.8 mm. The current at node *k* on ANF_*i*_ at time *t* with current spread is given by

$$I_{ANF_{k,i}}[t]=I_{E_{j}}[t]\cdot e^{\left(\frac{-d_{E_{j}ANF_{k,i}}}{\lambda }\right)}$$ (1)

where *λ* is the current attenuation distance constant (set to 2.784 to achieve a current decay of 3.12 dB/mm [27]); $I_{E_{j}}[t]$ is the current in the *j*th electrode *E*_*j*_ at time; and $d_{E_{j}ANF_{k,i}}$ is the distance between *E*_*j*_ and node *k* on *ANF*_*i*_. The case that was not considered the current from the neighboring electrode was defined as “without current spread.” The current at node *k* on ANF_*i*_ at time *t* without current spread was determined using only the current in the *i*^th^ electrode *E*_*i*_ at time *t*, as expressed by Eq 1.

An ANF model developed by Woo et al. [22], which included neural adaptation, was used to predict the ANF responses to the rippled noise stimuli. The model was based on the Hodgkin–Huxley model and its parameters were set based on the geometry for cats. It was composed of a peripheral axon with three internodes, the cell body, and a central axon with 20 internodes. Each Ranvier node included voltage-dependent sodium and potassium ion channels. The model utilized a Fox algorithm [28–30] that used noise variables to compute the numbers of active ion channels for the different types of ions. The transmembrane potential *V*_*m*_ in each axon compartment *k* at time *t* is given by

$$\begin{array}{l} -(\frac{V_{m}^{\left[k+1\right]}[t]-V_{m}^{\left[k\right]}[t]}{R_{a}^{\left[k+1,k\right]}}-\frac{V_{m}^{\left[k\right]}[t]-V_{m}^{\left[k-1\right]}[t]}{R_{a}^{\left[k,k-1\right]}})+C_{m}^{\left[k\right]}\frac{V_{m}^{\left[k\right]}[t+\Delta t]-V_{m}^{\left[k\right]}[t]}{\Delta t}+\frac{V_{m}^{\left[k\right]}[t]}{R_{m}^{\left[k\right]}}+I_{K}^{\left[k\right]}[t]+I_{Na}^{\left[k\right]}[t]\\ =\frac{V_{e}^{\left[k+1\right]}[t]-V_{e}^{\left[k\right]}[t]}{R_{a}^{\left[k+1,k\right]}}-\frac{V_{e}^{\left[k\right]}[t]-V_{e}^{\left[k-1\right]}[t]}{R_{a}^{\left[k,k-1\right]}} \end{array}$$ (2)

$$V_{e}^{\left[k\right]}[t]=I_{ANF_{k,i}}[t]\cdot \rho_{e}$$ (3)

where *R*_*m*_ is the voltage independent nodal resistance, *C*_*m*_ is the capacitance of the single-node model, *R*_*a*_ is the axoplasmic resistance, and *I*_*K*_ and *I*_*Na*_ are the ion current of potassium and sodium, respectively. The extracellular potential on node $k(V_{e}^{\left[k\right]})$ of ANF_*i*_ is calculated by multiplying the electric stimulus $I_{ANF_{k,i}}$ by the mean resistivity of the extracellular medium, *ρ*_*e*._ The values of the parameters in Eq 2 are listed in the S1 Appendix, while details of the ANF model are available in the work of Woo et al. [22, 25, 30]. The electrode–ANF distance was determined as the distance between the center of the stimulating electrode and the ANF surface (see Fig 1B). Although both the height and width of the Scala tympani are smaller than 2.0 mm, and the tympani is within 1.5 mm from the round window [31], the electrode–ANF distance in this study was varied between 0.23 and 2.08 mm to investigate the effect of an extremely large electrode–ANF distance on the SRD performance. Five specific distances were considered, namely 0.23, 0.68, 1.18, 1.88, and 2.08 mm, with the corresponding distances between the electrode surface and ANF being 0.05, 0.5, 1.0, 1.7, and 1.9 mm, respectively.

### 2.4 Graphical representation of an ensemble of ANFs responses

**Fig 3:**
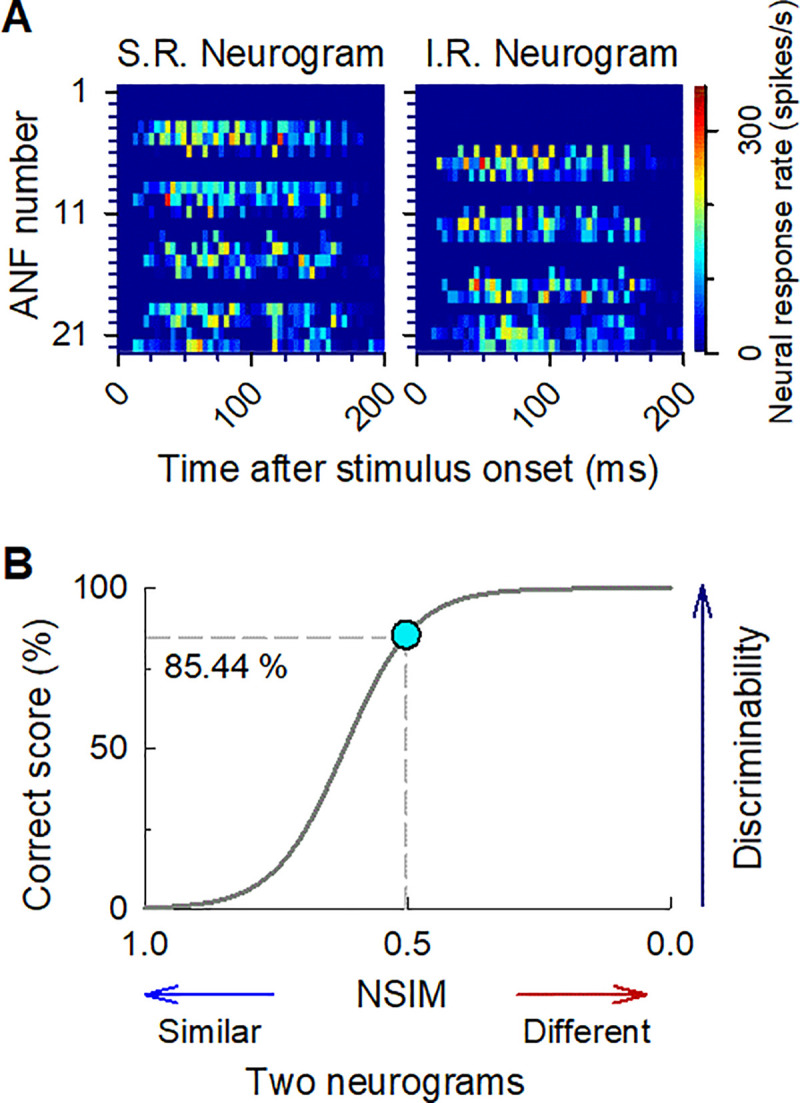
Procedure for calculating the correct score for spectral ripple discrimination. (A) Example neurograms for standard (left) and inverted (right) ripple noises with 1 r/o. In the neurograms, the differing extents of neural activity are depicted by a color scale, from 0 (blue) to 360 (red) spikes/s over time, which is represented by the horizontal axis. (B) Correct score function for converting the NSIM into the correct score. An NSIM of 0.5, which is comparable to that between the neurograms in (B), converts into a correct score of 85.44%.

Post-stimulus time histograms (PSTHs) simulated from 22 ANFs with a bin width of 4 ms were computed with ANF responses used to stimulate the pulses for each channel. The process was repeated 30 times and graphically represented by a neurogram. Fig 3A shows example neurogram responses to the standard and inverted ripple stimuli with ripple density of 1.0 r/o. The *y*-axis of the neurogram shows the ANF number corresponding to each electrode number (i.e., the frequency band), while the *x*-axis represents time with a bin of 4 ms. The color scale ranges from 0 (blue) to 360 (red) spikes/s.

### 2.5 Scoring spectral ripple discrimination performance

We calculated the similarity between the neurograms for the standard and inverted ripple noise stimuli using a neurogram similarity index measure (NSIM), with the purpose of predicting the SRD performance, as shown in Fig 3 [32, 33]. The NSIM between two neurograms “a” and “b” of the same size was computed by comparing the luminance and structure of the two neurograms. The luminance *l* was estimated as the mean intensity of each neurogram, and the structural similarity *s* was quantified using the correlation between the two neurograms. To calculate the NSIM, the neurogram intensity range of 0–360 spikes/s was normalized to 0–255. The NSIM was calculated as follows:

$$NSIM(a,b)=l(a,b)\cdot s(a,b)={\left(\frac{2\mu_{a}\mu_{b}+C_{1}}{\mu_{a}^{2}+\mu_{b}^{2}+C_{1}}\right)}^{\alpha }\cdot {\left(\frac{\sigma_{ab}+C_{2}}{\sigma_{a}\sigma_{b}+C_{2}}\right)}^{\gamma }$$ (4)

where *μ* and *σ* are respectively the mean and standard deviation of the 3 × 3 Gaussian-weighted window that moves pixel-by-pixel. At each point, the NSIM, which is based on the local statistics of relevant parameters (*μ* and *σ*), was calculated within the Gaussian-weighted window to obtain a NSIM map. The mean NSIM map was then used for the overall similarity index. The weighting parameters *α* and *γ* were set to 1, and *C*_*1*_ and *C*_*2*_ were respectively defined as (0.01*L*)^2^ and (0.05*L*)^2^, where *L* = 255, which is the intensity range of the neurograms.

The NSIM-based SRD performance was used to develop a correct score function based on the sigmoid decision rule, as follows:

$$correct\;score=\frac{1}{1+e^{-S(NSIM-D_{thr})}}\times 100$$ (5)

where *S* is the slope of the function, NSIM is the similarity between the standard and inverted ripple noise neurograms, and *D*_*thr*_ is the discrimination threshold. The discrimination threshold and the slope of the function were optimized by the mean of the percent-correct scores for some CI subjects, as calculated by Won et al. [6] from raw data. The median of the simulation percent-correct scores for the five electrode–ANF distances for each ripple density was compared with the corresponding mean for the CI subjects. The values that produced the smallest square root error between the median simulation percent-correct scores and the mean percent-correct scores for the CI subjects were chosen as the discrimination threshold and the slope of the function, namely 0.61 and 21, respectively.

The neurograms for the standard and inverted rippled noise stimuli are shown in Fig 3A. The phase reversal between the two rippled noise stimuli (standard and inverted) can be observed from the neurograms. Fig 3B shows the correct score functions based on a NSIM of 1.0 r/o. A smaller NSIM corresponds to a higher discrimination score, which take a value between 0% and 100%. The NSIM for 1.0 r/o is 0.5, which corresponds a correct score of 85.44%. The percent-correct score was determined by averaging the results for six repetitions. The percent-correct score for a human CI subject can be calculated using the number of correct responses for each ripple density, as was done by Won et al. [6]. The ACE strategy was used to compare the model-predicted percent-correct scores with the scores from 15 human CI subjects who used Nucleus CI24.

## 3. Results

### 3.1 Neural response to the pulse train

**Fig 4:**
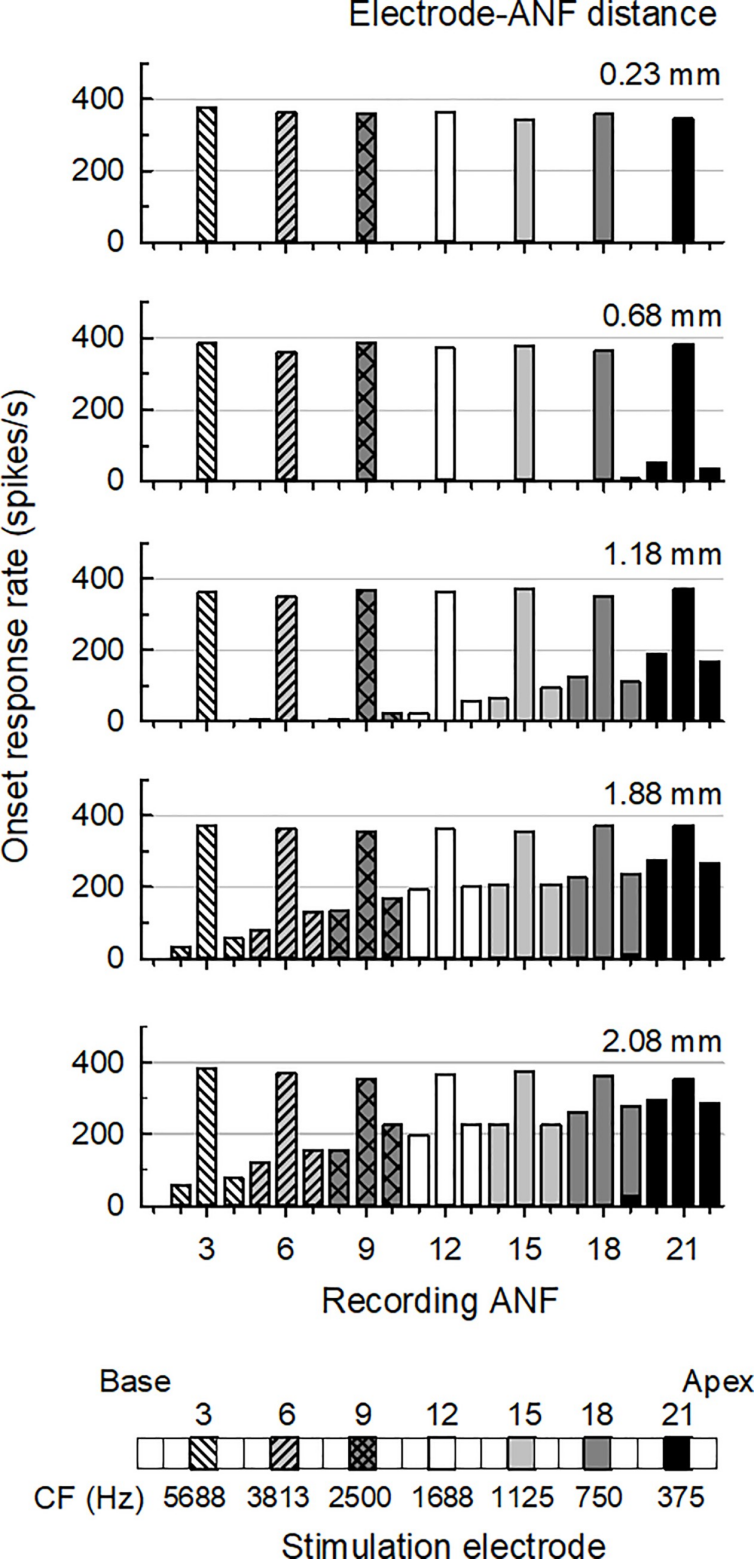
Spread of excitation separately simulated using seven channels (3rd, 6th, 9th, 12th, 15th, 18th, and 21st) at five electrode–ANF distances. Each pattern of the bar indicates an onset response rate recorded from 22 ANFs, one for each channel, having the same pattern as in the legend. The spacing between the ANFs in the apical region is narrower than that of the ANFs in the basal region, consistent with the trend of the electrode spacing.

Fig 4 shows the ANF responses for the five considered electrode–ANF distances, indicating the effect of the excitation spread. Each 900-Hz pulse train stimuli level was set to evoke the ANF response of 360 spikes/s. The channels corresponding to the 3rd, 6th, 9th, 12th, 15th, 18th, and 21st ANFs were selected and the simulations for each channel were performed separately. The ANF responses for each channel were expressed by combinations of seven colors and patterns, as shown in Fig 4. For an electrode–ANF distance of 0.23 mm, only the ANFs within each stimulation channel were evoked. However, responses were observed at the 20th and 22nd ANFs for an electrode–ANF distance of 0.68 mm, although the stimulation was only applied to the 21st channel. Because the spacing between the ANFs and electrodes was smaller at the apex, the excitation spread occurred only in the apical region. For an electrode–ANF distance of 1.18 mm, the excitation spread caused responses at the 9th, 12th, 15th, 18th, and 21st ANFs, which had relatively large spacing. Furthermore, at an electrode–ANF distance of 1.88 and 2.08 mm, additional spread of excitation was induced at the 3rd and 6th ANF. However, the spread of excitation was not observed in the basal region because the spacing between the ANFs was larger than at the apex. The spread of excitation was broader for larger distances between the electrodes and ANFs, and smaller when the electrodes were closer to the ANFs. The model revealed an increase in the spread of excitation with increasing electrode–ANF distance, consistent with previous findings [34, 35].

**Fig 5:**
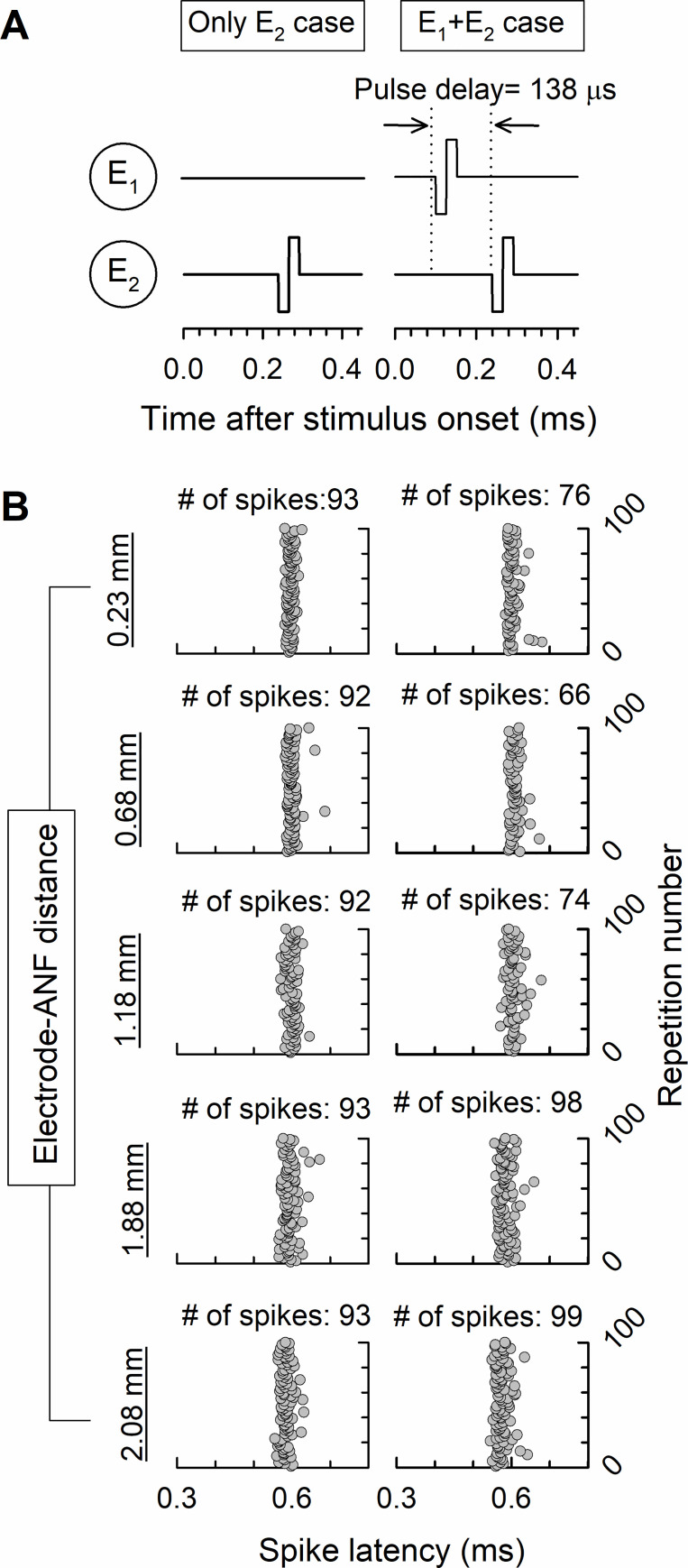
ANF response for the two-pulse paradigm. (A) Two cases, namely with and without a preceding pulse. The pulses were presented to electrodes 1 and 2, with the second presented 138 μs after the first for the E_1_+E_2_ case. (B) Dot raster plot of the neural activity for 100 repetitions. The left column represents the neural response to the stimulation pulse presented from only E_2_ (E_2_ case) while the right column represents the neural response to two pulses sequentially delivered from E_1_ and E_2_ (E_1_+E_2_ case). The ANF responses for the five considered electrode–ANF distances are shown top-to-bottom.

The stimulation pulses were non-simultaneously delivered to the eight elected channels during each cycle using the ACE strategy, but the stimulation spread to other ANFs than intended. The undesirable pulses caused channel interaction, which resulted in spectral smearing, and ultimately poor SRD performance. Fig 5 shows the ANF response with the two-pulse paradigm for five different electrode–ANF distances. Two cases were considered in the simulation of the ANF responses to investigate the channel interaction with respect to the electrode–ANF distance. In one case, the stimulation pulse was delivered from only one channel, and in the other case, two stimulation pulses were sequentially delivered to two channels, as shown in Fig 5A. Electrodes 1 and 2 were used to present biphasic pulses with a duration of 25 μs, and the ANF response was measured using the 2nd ANF under the 2nd electrode. The stimulation pulse level was set to the current level, which evoked neural firing of 90 spikes during 100 repetitions. The onset of the second pulse was delayed to 138 μs from that of the first. The pulse delay of 138 μs corresponded with the pulse rate of 7200 pps, which produced 900 pps for a single channel. The number of spikes in the response to the stimulation pulse from only E_2_ was about 90 for all the considered electrode–ANF distances. However, a change in neural activity could be observed for the E_1_+E_2_ case. The neural activity for the second pulse was inhibited for electrode–ANF distances of 0.23, 0.68, and 1.18 mm, while that for distances of 1.88 and 2.08 mm increased. There was no neural activity in response to the first pulse. These findings indicate that the pulses from neighboring channels inhibited or facilitated neural activity, and these differential channel effects could be observed with respect to the electrode–ANF distances.

### 3.2 Neural response to spectral-ripple stimuli

**Fig 6:**
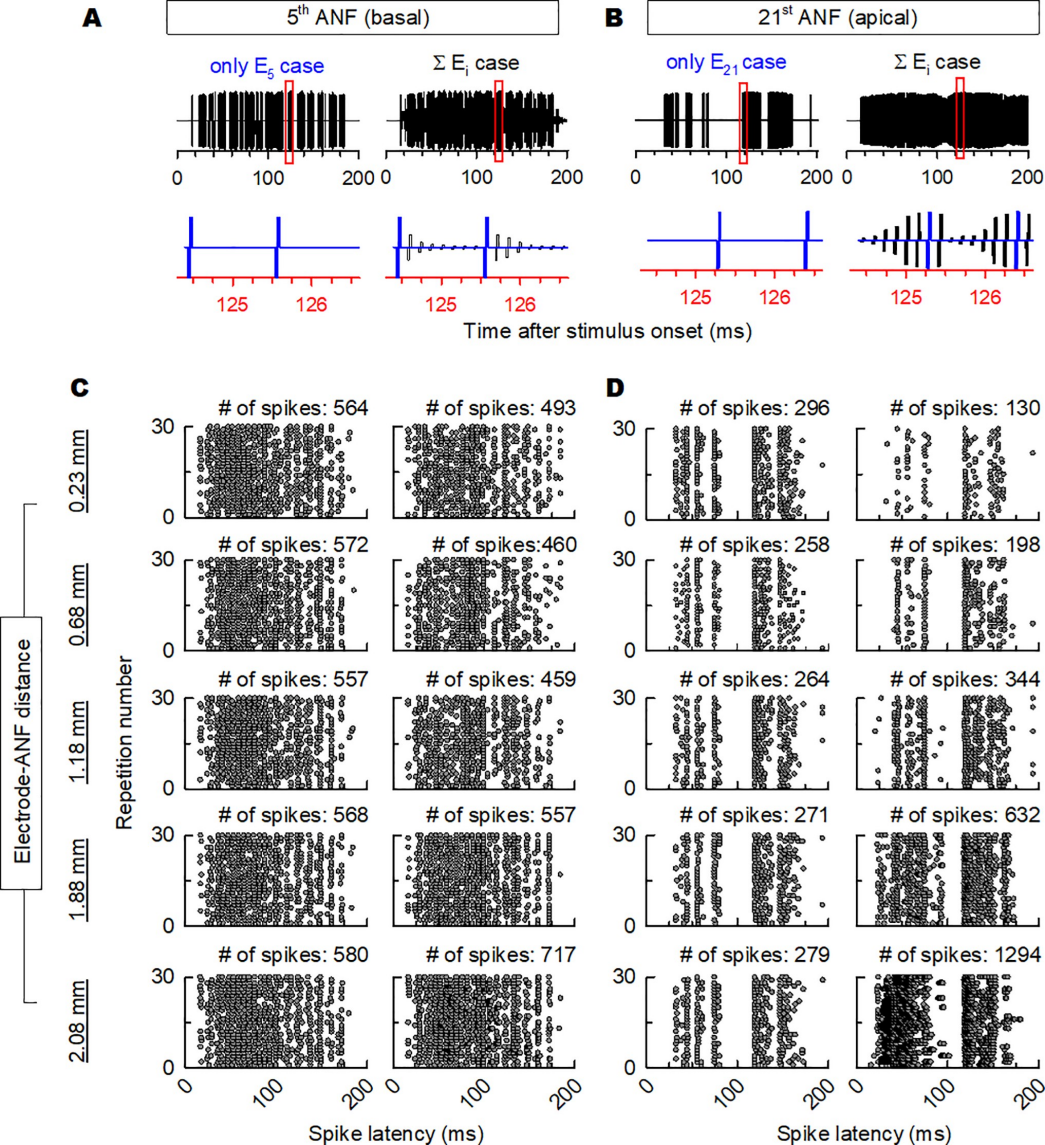
Example of the stimulation pulses and dot raster plot of the spike activity for 30 repetitions over the stimulus time for the 5th ANF in the basal region and 21st ANF in the apical region. Panels A and B show the stimulation pulses over a time interval defined by a window of 200 ms and a window of 124–127 ms, respectively (red square in the stimulation pulses of 200 ms). The left columns of panels A and B present the stimulation pulses from only the 5th and 21st channels, respectively (blue line in detailed stimulation pulses), while the right columns of panels A and B show the stimulation pulses including those from the neighboring channels due to current spread (black line in detailed stimulation pulses). Panels C and D show the spike activity for each stimulation in panels A and B, respectively.

Fig 6 shows the effect of the pulses delivered from neighboring channels on the ANF responses for a standard ripple noise with a ripple density of 1.0 r/o. The stimulation pulses of the 5th channel (basal region) and the 21st channel (apical region) are illustrated in Fig 6A and 6B, respectively, while the ANF responses to the stimulation pulses are shown in Fig 6C and 6D. The left column of each panel shows the stimulation pulses and the ANF response to the primary channel (only the *i*^th^ channel) without current spread, while the right column of each panel shows the combined stimulation pulses of both the primary channel and the neighboring channels, taking into consideration the effect of the current spread. The ANF responses were measured for all the five electrode–ANF distances. The stimulation pulses of the primary channels (5th and 21st channels) were positioned based on the stimulation order (basal to apical). The pulse of the 5th channel was presented earlier than the other stimulation pulses, while that of the 21st channel appeared later than those of the other stimulation pulses.

The neural activity for the stimulation pulses from one channel and the combined stimulation pulses are presented in the dot plots in Fig 6C and 6D. An examination of the left columns in these figures reveals similar numbers of spikes of the stimulation pulses from a single channel for the five electrode–ANF distances, while the number of spikes for the combined stimulation pulses increases with increasing electrode–ANF distance because of the large spread of excitation. We observed that the ANF responses to combined stimulation pulses were smaller than those to stimulation by only the 5th channel for electrode–ANF distances of 0.23–1.88 mm. These results indicate that the pulses delivered from the neighboring channels inhibited the ANF response. In contrast, for an electrode–ANF distance of 2.08 mm, the ANF responses to combined stimulation pulses were greater than those to the pulse train using only the 5th channel. In addition, as shown in Fig 6D, the ANF responses to the 21st channel were slightly different from those to the 5th channel. Although the ANF responses to combined stimulation pulse trains were inhibited for electrode–ANF distances of 0.23–0.68 mm, they were facilitated for electrode–ANF distances of 1.18–2.08 mm. While the stimulation pulses for neighboring channels inhibited the ANF responses for shorter electrode–ANF distances, the stimulation pulses delivered from the neighboring channels for large electrode–ANF distances facilitated greater ANF responses.

**Fig 7:**
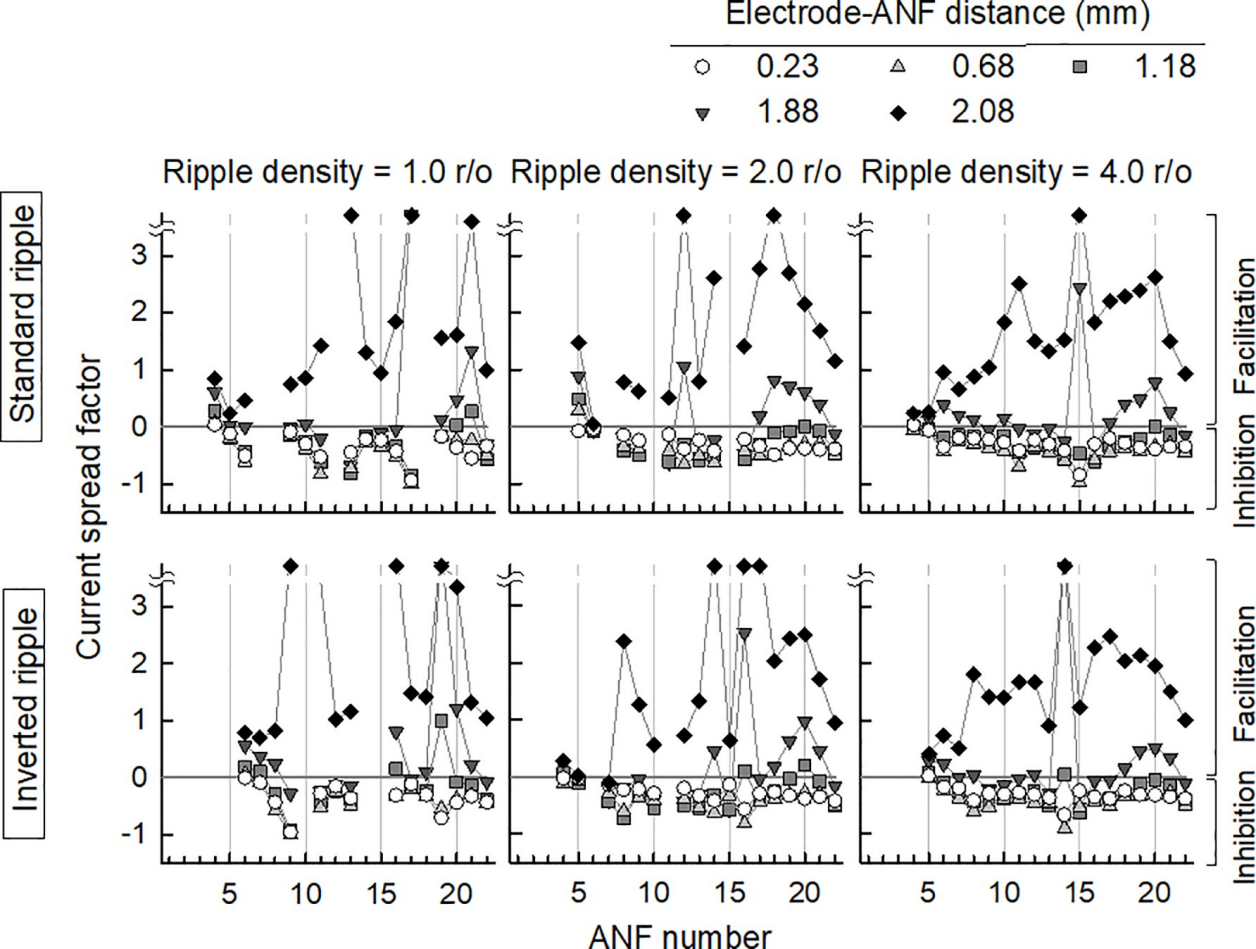
Current spread factors for three ripple densities and five electrode–ANF distances. The upper and bottom panels are respectively for standard and inverted ripple noises, while the left, middle, and right columns are for ripple densities of 1.0, 2.0, and 4.0 r/o, respectively. The gray line, which is the current spread factor of zero in each graph, indicates no effect of the current spread. A positive current spread factor indicates that a pulse delivered from a neighboring channel facilitates the excitation of the neural response, whereas a negative current spread factor indicates inhibition of the neural response. For an electrode–ANF distance of 2.08 mm, current spread factors above 3.5 are equally represented.

The effect of the current spread on the ANF responses could be predicted by analyzing the differences between the numbers of spikes when using the current spread modeling with that when not using the modeling. Fig 7 shows the current spread factors calculated by the difference between the total numbers of spikes with the current spread and without the current spread, divided by the total numbers of spikes without the current spread. The current spread factor is equal to zero if there is no effect on the ANF response to the primary channel. A negative current spread factor indicates inhibition of the ANF response to the primary channel, whereas a positive current spread factor indicates facilitation of the ANF response. The *x*-axis in Fig 7 represents the ANF number for which a neural response is simulated, while the *y*-axis represents the current spread factor for the ANF responses. The current spread factors for the five considered electrode–ANF distances are distinguished by the five different patterns, with the left, middle, and right columns corresponding to ripple densities of 1.0, 2.0, and 4.0 r/o, respectively. The upper and lower panels in Fig 7 represent the current spread factors for the standard and inverted ripple stimuli, respectively. The stimulated ANFs for the standard ripple noise overlap those for the inverted ripple noise owing to the narrow ripple spacing for a ripple density of 4.0 r/o.

Overall, the ANF responses to stimulation electrodes located at 0.23–1.88 mm had smaller current spread factors due to inhibition by the preceding pulses. However, the current spread factors were positive in the apical region for electrode–ANF distances of 1.18–1.88 mm because the pulses from neighboring channels facilitated the ANF response. At the largest electrode–ANF distance of 2.08 mm, the current spread factors were positive in all regions, with some exceeding 3.5 through facilitation by the large electrode–ANF distance. More than half of the pulses from the neighboring electrodes induced spike activity. This indicates that the effect of the channel interaction induced by neighboring channels increases the ANF response for a larger electrode–ANF distance and narrower electrode spacing.

### 3.3 Predicted SRD performance

**Fig 8:**
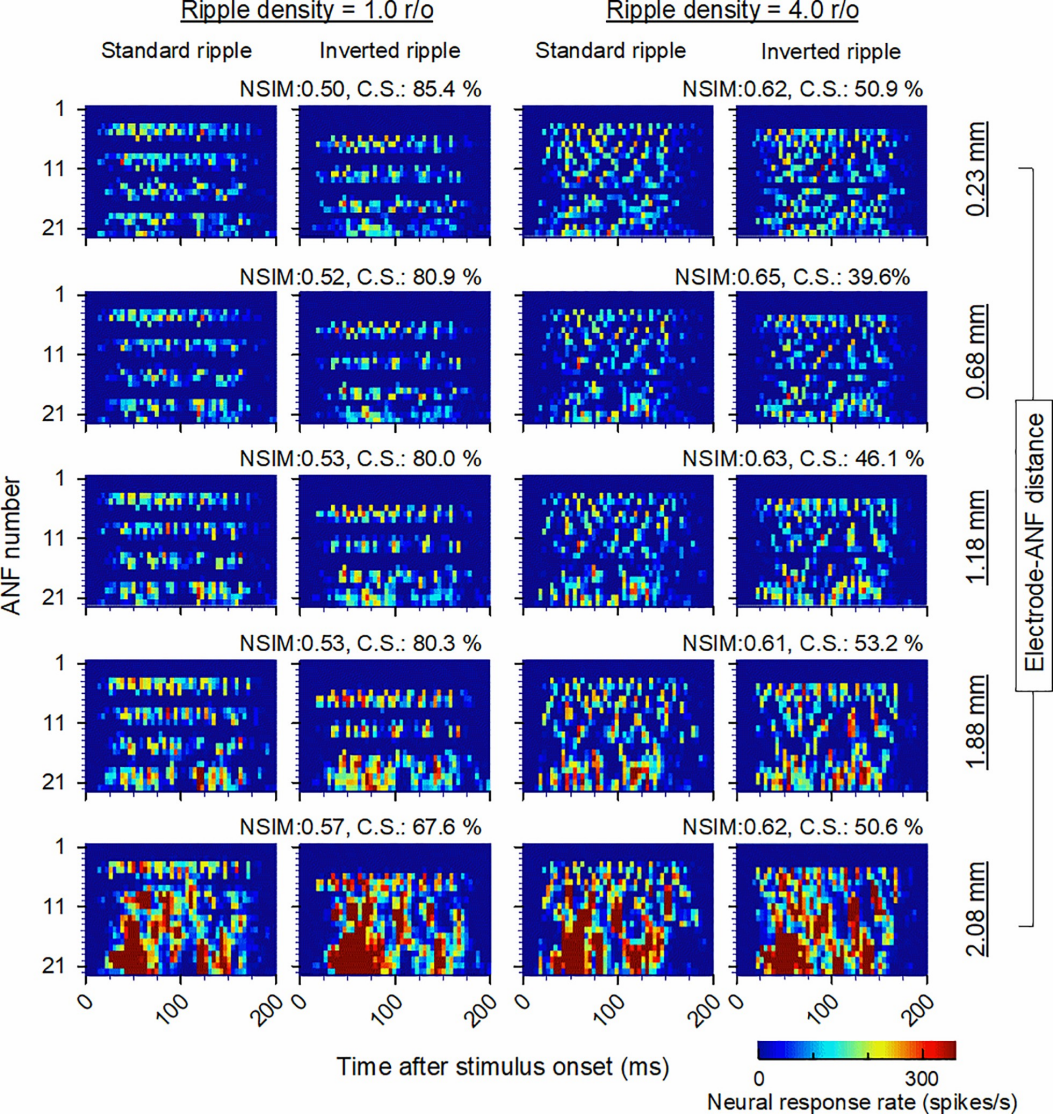
Example neurograms for ripple densities of 1.0 (columns 1 and 2) and 4.0 r/o (columns 3 and 4) and five electrode–ANF distances of 0.23, 0.68, 1.18, 1.88, and 2.08 mm (top to bottom). The indicated NSIMs and correct scores (C.S.) were computed using the neurograms for the standard and inverted ripple noises.

Fig 8 shows the neurograms for the standard and inverted ripple noises with ripple densities of 1.0 and 4.0 r/o and the five considered electrode–ANF distances. For both ripple densities at for electrode–ANF distances of 0.23–0.68 mm, the spread of excitation was partial in the apical region where the ANF spacing was narrow. However, for electrode–ANF distances of 1.18–2.08 mm, the spread of excitation occurred in both the basal and apical regions. As can be observed from Fig 8, for an electrode–ANF distance of 0.23 mm and ripple density of 1.0 r/o, the numbers of ANFs that respond to the standard and inverted ripple noises significantly differ. The responses to the standard ripple noise were dominant for the 5th, 10th, 15th, and 19th ANFs, whereas the 7th, 12th, 17th, and 21st ANFs responded strongly to the inverted ripple noise. However, for an electrode–ANF distance of 2.08 mm, the variously dominant ANF responders between the two ripple noises were observed in the basal region of the electrode array, and not in the apical region, owing to the current spread. The NSIM for an electrode–ANF distance of 0.23 mm was 0.5, while it was for an electrode–ANF distance of 2.08 mm, which is 0.57 higher. These NSIMs correspond to correct scores of 85.4% and 67.6%, respectively, as determined by the sigmoid function in Fig 3B. For a ripple density of 4.0 r/o, the dominant ANF responders for the standard and inverted ripple noises overlapped, even for a short electrode–ANF distance of 0.23 mm. The NSIM was relatively high at 0.62 with a corresponding correct score of 50.9%, which is lower than that for a ripple density of 1.0 r/o. The current spread had no significant effect on the similarity between the two neurograms for a ripple density of 4.0 r/o and a large electrode–ANF distance.

**Fig 9:**
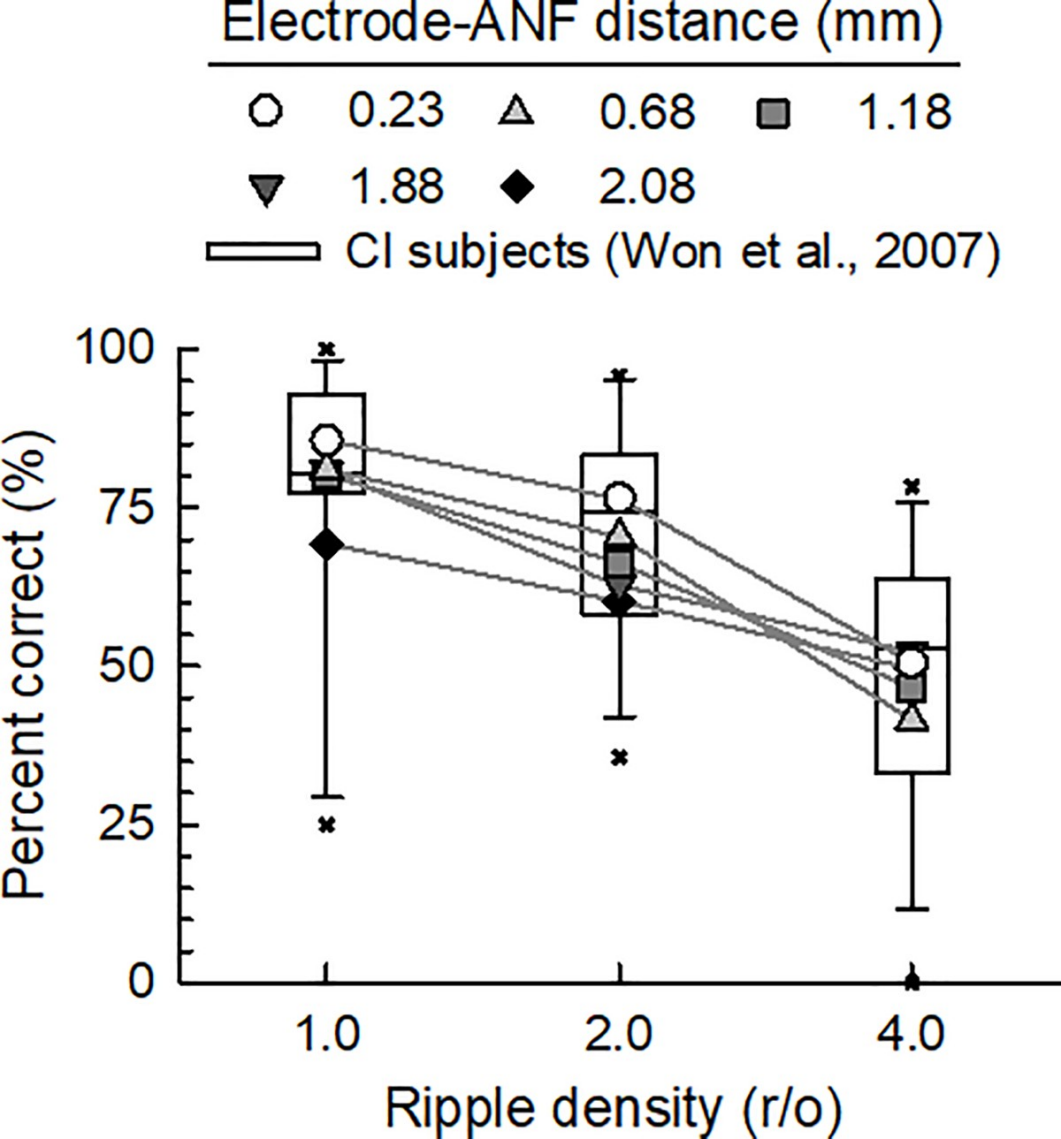
Effect of the electrode–ANF distance on the SRD performance. Each box plots the median, 10th, 25th, 75th, and 90th percentiles of the data of Won et al. (2007) [6] for 15 CI subjects as obtained by the ACE strategy, with the asterisks indicating the outliers. The results obtained by the computational model for the five electrode–ANF distances are indicated by the five different symbols, covering between the 25th and 75th percentiles of the CI subject data for ripple densities of 1.0 and 2.0 r/o.

In Fig 9, the white square boxes indicates the percent-correct scores for human CI subjects determined by the ACE strategy of Won et al. (2007) [6]. The lines of the box respectively indicate the 10th percentile, 25th percentile, median, 75th percentile, and 90th percentile from the top. The percent-correct score decreased with increasing ripple density, although there were large across-subject variabilities for ripple densities of 1.0 (mean = 77.59, SD = 21.42), 2.0 (mean = 71.46, SD = 16.72), and 4.0 r/o (mean = 48.34, SD = 20.64), as shown in Fig 9. Further, the percent-correct score determined by the computational model decreased with increasing ripple density for all the electrode–ANF distances, and decreased with increasing electrode–ANF distance for 1.0 and 2.0 r/o. It is noteworthy that the predicted percent-correct scores almost covered the range between the 25th and 75th percentiles of the clinical data, demonstrating that the electrode–ANF distance is a potential source of across-subject variability in the SRD performance. However, at 4.0 r/o, the predicted percent-correct scores covered a narrow range of the clinical data.

## 4. Discussion

In this study, the spread of excitation and channel interaction with respect to the electrode–ANF distance in a CI was investigated using a computational neural model. We found that the SRD performance was influenced by not only the spread of excitation, but also the differential neural excitability induced by channel interaction with respect to the electrode–ANF distance, although the electrodes were non-simultaneously activated.

As can be observed from the results in Fig 5, the neural excitation spreads out with increasing electrode–ANF distance, consistent with previous findings [35, 36]. Through simulation of neural activation, Goldwyn et al. (2010) demonstrated that stimulation with a smaller electrode–neuron distance produced a narrower spread of excitation [35]. In another study, DeVries et al. (2016) measured the electrically evoked compound action potential (ECAP) for CI subjects and showed that the width of the ECAP was positively correlated with the electrode-to-modiolus distance [36]. The results of the present study agree with those of these earlier studies.

The current modeling study showed that the channel interaction variously affected the neural excitation in response to ripple noise stimuli. This is due to the level of the pulses from neighboring channels increasing with increasing electrode–ANF distance. The low-level pulses that are delivered from the neighboring channels for a short electrode–ANF distance inhibit the ANF responses, whereas the responses are facilitated by the high-level pulses delivered from the neighboring channels for longer electrode–ANF distances. Applying cat data to a model, Dynes (1996) [19] showed that a sub-threshold pulse could inhibit the neural response to a subsequent pulse. However, the exact biophysical mechanisms of the inhibition remained unclear. Frankenhaeuser and Vallbo (1965) [37] suggested that residual sodium inactivation might be the reason for the infrequent response of neuron spikes to pulse train stimuli. However, this does not sufficiently explain the inhibition of neural activity for a subthreshold pulse. The facilitation of ANF response for a longer electrode–ANF distance results from charge accumulation sufficient to evoke neural spiking. However, the SRD performance was observed to depreciate with increasing electrode–ANF distance owing to the large spread of excitation and channel interaction. Nevertheless, the SRD performance could be influenced by other factors. Zhou (2017) suggested that the deactivation of some stimulation electrodes with poor low-rate detection thresholds could be used to improve the SRD performance [38].

We simulated 22 ANFs to observe the SRD performance with respect to the electrode–ANF distance, which was set to five different values between 0.23 and 2.08 mm. However, the employed model has some limitations. First, the model is unrealistic because of its small number of ANFs and the applied cat cochlear morphology. Histological studies have shown that human and cat ANFs differ in terms of the number of myelinated segments in the peripheral process, cell body area, and myelinated thickness of the cell body morphology, which is very similar to that of the human cochlear [39, 40]. While the morphometric measures [41] and spiking behavior of cat ANFs in response to a single electric pulse or electric pulse train [42, 43] have been systematically investigated, rarely have human ANFs been similarly explored. Thus, a cat ANF model validated against physiological data can be flexibly used to explore and predict neural activities in the cochlear neuron of other species. The spread of excitation with the pulse train can be investigated as shown in Fig 4. The number of ANFs used in this study was thus sufficient to observe the SRD performance. Although the detailed neural response can be obtained using a larger number of ANFs, this would not significantly impact the SRD performance.

A second limitation of the model used in this study is that the electrode–ANF distance was uniformly set from the apex to the base, whereas the diameter of the scala tympani reduces from the base to the apex. Thirdly, an individualized model incorporating subject-specific information such as the actual electrode–ANF distance of the CI user is required to precisely predict the SRD performance. Such individualization was not considered in our model, and not only the CI performance, but also the neural excitation patterns for the various considered conditions were impacted. This limitation of the current model can be addressed by incorporating a broader population of fibers with 3D cochlear modeling [44]. Lastly, considering that a ripple density of 4.0 r/o was observed to be too high for the filter-bank used in the ACE strategy employed in this study, resulting in inability to fully resolve the ripple spectra, it is possible that human CI users actually adopt a loudness cue rather than explicit focus on a spectral cue. This perceptual mechanism was not effectively implemented in the present model, and this might have affected the discrepancy between the human and model data. While it was proper for us to use amplitude modulation of the stimulus in the frequency domain to evaluate the effect of the electrode–ANF distance on the SRD performance, modified SRD tests that consider temporal resolution have been developed to eliminate some confounds of the traditional SRD test used in this study [45, 46]. The inhibition and facilitation of the neural responses induced by channel interaction also influenced the temporal resolution in the present study. Spectral-temporal discrimination tests can be used to investigate the effect of the electrode–ANF distance on both the spectral and temporal resolutions in a CI.

Despite the above limitations of the model used in this study, the application of a computational modeling approach has some advantages. Because it enables simulation of the anatomical variations resulting from degeneration of ANF, such as the demyelination and loss of spiral ganglion cells, it facilitates evaluation of the effects of various factors on the SRD performance, especially when the model is individualized through the incorporation of anatomical variations. In addition, the utilization of cathodic-first biphasic pulses to stimulate ANFs enabled more focused positional stimulation compared with the use of an anodic pulse, as demonstrated by Rattay and Aberham [47]. However, the findings of many studies have suggested a polarity effect in degenerated ANFs [48, 49]. A computational model can be used to investigate the effect of the stimulation strategy (pulse polarity, pulse shape, or stimulation rate) on the SRD performance.

A poor SRD performance was determined to be correlated with not only the spread of neural excitation, but also with channel interaction due to neural excitation when using a long electrode–ANF distance. Considering the relationship between the electrode position and speech perception [12, 36, 50], it can be inferred that speech perception may also be affected by neural excitability under channel interaction. Nevertheless, channel interaction and the resultant inhibition or facilitation of neural response occurs regardless of the electrode–ANF distance. Hence, reduction of the spread of excitation by adopting a shorter electrode–ANF distance can be used to improve the SRD performance, It can thus be suggested that a perimodiolar electrode that curves toward the modiolus to reduce the distance between the electrode and the nerve may enhance SRD performance and speech intelligibility. In view of the forgoing, reduction of channel interaction under non-simultaneous stimulation would be natural next area for further study.

To summarize, we used a computational ANF model to examine the effect of channel interaction with respect to the electrode–ANF distance on the SRD performance in a CI. The results indicate that channel interaction variously impacts neural excitability depending on the electrode–ANF distance, and that the positions of the electrodes within the cochlea account for across-subject variability of the SRD performance. The employed model promises to contribute to the development of effective strategies for improving the speech perception of CI users through reduction of current spread.

## Supporting information

S1 Appendix (DOCX)
