# Supplementary material for: A computational study to model the effect of electrode-to-auditory nerve fiber distance on spectral resolution in cochlear implant
Source: PLoS One. 2020 Aug 3;15(8):e0236784. doi: 10.1371/journal.pone.0236784 (PMC7398541; doi:10.1371/journal.pone.0236784)
Supplement: S1 Appendix — (DOCX) [file pone.0236784.s001.docx]

**Appendix**

The ANF model used in this study was developed by Woo et al. [22,25,30]. Table 1 in this appendix contains the values of the model parameters in Eqs. 2 and 3.

Table 1. Electrophysiological parameters of the ANF model used in this study

| Parameter | Value | | |
| --- | --- | --- | --- |
| **Node of Ranvier** |  | | |
| Resistance, $R_{m}$ | 166.2 kΩmm^2^ | | |
| Capacitance, $C_{m}$ | 0.5125 nF/mm^2^ | | |
| **Internode** |  | | |
| Resistance, $r_{m}$ | $r_{m}=\frac{\rho_{m}(D-d)}{2\pi dl_{\varepsilon}}$ ^a^ | | |
| Capacitance, $c_{m}$ | $c_{m}=\frac{2\pi\varepsilon_{0}\varepsilon_{r}l_{\varepsilon}}{\ln(D/d)}$ | | |
| **Na channel** |  | | |
| Nernst potential, $E_{Na}$ | 74 mV | | |
| Density, $\rho_{Na}$ | 80/μm^2 b^ | | |
| Conductance, $\gamma_{Na}$ | 22.65 pS | | |
| **K channel** |  | | |
| Nernst potential, $E_{K}$ | -88 mV | | |
| Density, $\rho_{K}$ | 45/μm^2 b^ | | |
| Conductance, $\gamma_{K}$ | 50 pS | | |
| **Na^+^ gating kinetics** | A | B | C |
| *$\alpha_{m}$* | 1.872 | 25.41 | 6.06 |
| *$\beta_{m}$* | -3.793 | 21.001 | 9.41 |
| *$\alpha_{h}$* | -0.549 | -27.74 | 9.06 |
| *$\beta_{h}$* | 22.57 | 56 | 12.5 |
| **K^+^ gating kinetics** |  |  |  |
| *$\alpha_{n}$* | 0.129 | 35 | 10 |
| *$\beta_{n}$* | -0.324 | 35 | 10 |
| **Axoplasmic resistance, $R_{a}$** | 6378 Ωmm | | |
| **Resting potential, $E_{rest}$** | -78 mV | | |
| **Extracellular resistivity, $\rho_{e}$** | 0.3 kΩcm | | |

^a^ $\varepsilon_{0}$ is the permittivity of free space ($8.854\times{10}^{-12}$ F/m), $\varepsilon_{1}$ is the myelin dielectric constant (1.27), $I_{\varepsilon}$ is the length of compartment 1 of the nine sub-units of the internode, D is the outer myelinated fiber diameter, *d* is the unmyelinated (inner) fiber diameter, and $\rho_{m}$ is the intermodal resistivity (29.26 GΩmm).

^b^ Modified from $\rho_{Na}=27/\mu m^{2}$ and $\rho_{Na}=10.6/\mu m^{2}$ to achieve an absolute refractory period of 0.41 ms.

^c^ $\alpha_{m}, \alpha_{n}, \alpha_{h}, \beta_{m}, \beta_{n}=A(V-B)/\{1-\exp[\left( B-V \right)/C]\}, \beta_{h}=A/[1+exp\{{(B-V)}/{C\}]}$.
